# Combination Therapy Strategy of Quorum Quenching Enzyme and Quorum Sensing Inhibitor in Suppressing Multiple Quorum Sensing Pathways of *P*. *aeruginosa*

**DOI:** 10.1038/s41598-018-19504-w

**Published:** 2018-01-18

**Authors:** July Fong, Chaodong Zhang, Renliang Yang, Zhao Zhi Boo, Soon Keat Tan, Thomas E. Nielsen, Michael Givskov, Xue-Wei Liu, Wu Bin, Haibin Su, Liang Yang

**Affiliations:** 1 School of Biological Sciences, Nanyang Technological University, 60 Nanyang Drive, 637551 Singapore, Singapore; 2 Nanyang Environment and Water Research Institute (NEWRI), Interdisciplinary Graduate School, Nanyang Technological University, 50 Nanyang Avenue, 639798 Singapore, Singapore; 3 NTU Institute of Structural Biology, Nanyang Technological University, EMB 06-01, 59 Nanyang Drive, 636921 Singapore, Singapore; 4 School of Civil and Environmental Engineering, Nanyang Technological University, 50 Nanyang Ave, 639798 Singapore, Singapore; 5 Singapore Centre for Environmental Life Sciences Engineering (SCELSE), Nanyang Technological University, 60 Nanyang Drive, 637551 Singapore, Singapore; 6 Costerton Biofilm Center, Department of Immunology and Microbiology, University of Copenhagen, 2200 København N, Copenhagen, Denmark; 7 School of Physical & Mathematical Sciences, Nanyang Technological University, 21 Nanyang Link, 637371 Singapore, Singapore; 8 School of Materials Science and Engineering, Nanyang Technological University, 50 Nanyang Avenue, 639798 Singapore, Singapore

## Abstract

The threat of antibiotic resistant bacteria has called for alternative antimicrobial strategies that would mitigate the increase of classical resistance mechanism. Many bacteria employ quorum sensing (QS) to govern the production of virulence factors and formation of drug-resistant biofilms. Targeting the mechanism of QS has proven to be a functional alternative to conventional antibiotic control of infections. However, the presence of multiple QS systems in individual bacterial species poses a challenge to this approach. Quorum sensing inhibitors (QSI) and quorum quenching enzymes (QQE) have been both investigated for their QS interfering capabilities. Here, we first simulated the combination effect of QQE and QSI in blocking bacterial QS. The effect was next validated by experiments using AiiA as QQE and G1 as QSI on *Pseudomonas aeruginosa* LasR/I and RhlR/I QS circuits. Combination of QQE and QSI almost completely blocked the *P. aeruginosa las* and *rhl* QS systems. Our findings provide a potential chemical biology application strategy for bacterial QS disruption.

## Introduction

The emerging threat of antibiotic resistant bacterial pathogens has called for alternative strategies that could replace the usage of current antibiotics and minimize the development of resistance mechanism. One such strategy is to interfere with the bacterial signaling pathways governing the social behaviors involved in pathogenesis and drug-resistant biofilm formation^1^. Microbial organisms exhibit social behaviors and communicate with each other through quorum sensing (QS)^2–4^. By synthesizing small signal molecules, they respond collectively to regulate expression of virulence factors, biofilm development, secondary metabolite production, interactions with host and other microbes in a population-density dependent manner^5^. Targeting QS mechanisms has been put forward as an attractive approach to conventional infection control^1^.

Acylhomoserine lactone (AHL)-based QS signals are found in more than 70 bacterial species, in which many of them are pathogens^3,6^. In most cases, the structures of the AHLs are conserved with a homoserine lactone (HSL) ring connected to an acyl group with different chain length (n = 4–16)^5,7^. Multiple AHL-based QS systems often co-exist in individual bacterial species. There are two AHL-mediated QS systems in the opportunistic pathogen *Pseudomonas aeruginosa*, comprising the Lux homologues LasRI and RhlRI. LasRI and RhlRI function in a hierarchical manner in controlling the gene expression. LasI and RhlI are responsible for the synthesis of *N*-(3-oxododecanoyl) homoserine lactone (3-oxo-C12-HSL) and *N-*butanoylhomoserine lactone (C4-HSL) respectively, while the LasR and RhlR function as receptors for 3-oxo-C12-HSL and C4-HSL and subsequently activate gene expression of QS target genes^8–10^. On top of that, there is also a third signaling molecule “pseudomonas quinolone signal” (PQS) which is intertwined between the *las* and *rhl* systems^11^. QS defective *P. aeruginosa* mutants have much reduced virulence as compared to the wild-type strain and are unable to establish infections in several animal models^1,12,13^.

The concept of QS disruption is important not just in medicine and healthcare settings, but also in industrial membrane bioreactors, aquaculture and crop production^5,14^. It could be achieved by interfering with the QS signaling pathways (signal generator or receptor), or intercepting with the signal molecules (AHL)^15–17^. Enzymes that inactivate QS signals are called quorum quenching enzymes (QQE), while chemicals that disrupt QS pathways and reduce the expression of QS-controlled genes are called quorum sensing inhibitors (QSI)^5^. The first study on how a quorum quenching enzyme could be used to control bacterial infections was demonstrated by Dong *et al*.^18^. The enzyme encoded by *aiiA* gene isolated from Gram-positive *Bacillus* species is capable of inactivating AHL signals through hydrolysis of the ester bond of the homoserine lactone ring and quench QS signaling. It was proposed that the AHL-lactonase (AiiA) paralyzes QS signals and virulence factor production, hence allowing the host defense mechanisms to halt and clear the bacterial infection^19^.

Mathematical modeling has been a useful tool to answer basic and conceptual research questions in microbial physiology. In the last decade, mathematical modeling of QS has provided understanding to key components of QS networks^20^. It has been used to examine *P. aeruginosa* LasR/I circuit and predict the biochemical switch between two steady states of system (low and high levels of signal perception) and QS response to colony size and cell density^21^. In another study, Magnus *et al*. included both LasR/I and RhlR/I circuits of *P. aeruginosa* in their model. Their results suggested Vfr increases the affinity between LasR-AHL dimer and LasR promoter, which was supported by experiments showing that Vfr was important at initial but not later stages of QS induction^22^. Goryachev *et al*. analyzed *Vibrio fischeri* QS and found that dimerization of LuxR-AHL is important for the stability of QS network^23^. Altogether, the models developed in these studies provide a basic understanding of QS networks utilizing the LuxIR regulatory system and its homologues, which are identified in many Gram-negative bacteria^24,25^.

In this study, we explored the concept of combining QQE and QSI to disrupt both *las* and *rhl* AHL signaling and signal reception capacities, and reduce the pathogenicity of *P. aeruginosa*. The presence of multiple QS systems in individual bacterial species poses a challenge in QS interference strategy since multiple QS systems might have different induction dynamics during the bacterial growth. The two AHL-QS systems in *P. aeruginosa* are also highly adaptable and capable of responding to changing environmental stress conditions^26,27^. Combinational therapy could provide multiple points of attack to broaden the coverage and completely block the QS systems, which could significantly attenuate the survival of *P. aeruginosa* under stress conditions^28^. The two classes of QS disrupting agents have been studied independently, each with their own advantages and drawbacks. Small molecules as QSIs have well-known chemical structures, which in turn would allow structural activity and relationship (SAR) study and biological activity modification (i.e. pharmacodynamics and pharmacokinetics properties). The molecules can also diffuse into the cells and target the receptors, in contrast to QQE that act extracellularly to degrade AHLs^29^. For this study, we used G1 as QSI, which is a specific inhibitor to the LuxR-type receptor of *P. aeruginosa*^30^. Because of their distinct molecular structures and functional mechanisms, it would be interesting to explore the possible combination effect of QQE and QSI molecule in suppressing multiple *P. aeruginosa* QS pathways.

## Results

### Mathematical modeling shows combination effects between QQE and QSI on LasR/I circuit

**Figure 1:**
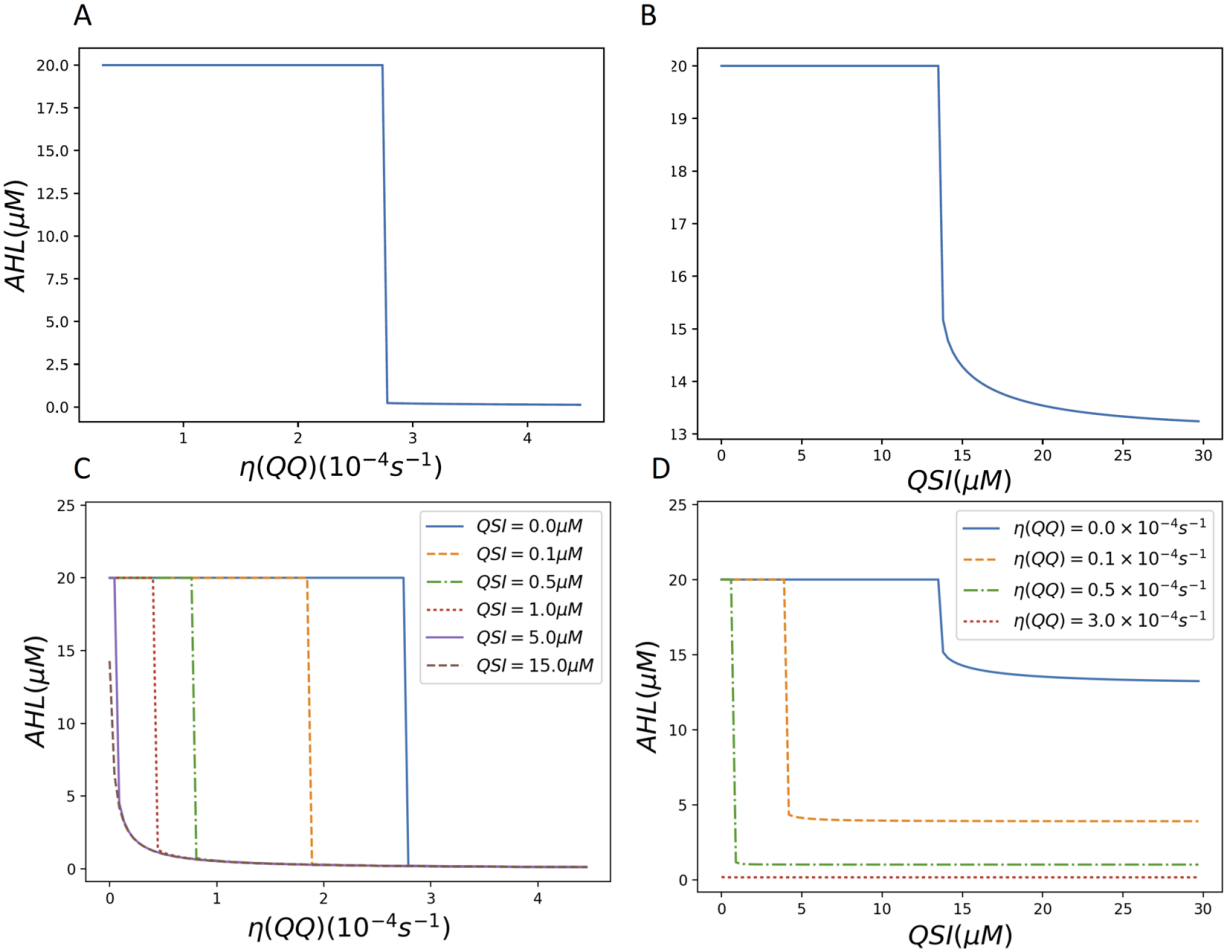
Simulation results of stationary AHL concentration to QQ and QSI. (**A**) η(QQ), (**B**) QSI, (**C**) *η*(QQ) at different QSI concentrations, and (**D**) QSI at different *η*(QQ) values.

When only QQE was present, simulation results showed a switching behavior between two steady states of low and high levels of AHL concentration (Fig. 1A). The AHL degradation rate by QQE is represented by *η*(QQ). When QQE concentration is low and *η*(QQ) is small, the stationary AHL concentration is high. However, when *η*(QQ) exceeds a threshold (2.8 × 10^−4^*s*^−1^, the stationary AHL concentration suddenly decreases to an insignificant value. Similar switching behaviors have been observed in the simulation response curves of QS components to population size^31^, cell volume fraction^21^, or to external AHL concentration^32^. Switching behaviors of QS networks have also been observed experimentally at individual cell level^33,34^.

When only QSI was present, similar switching behavior was observed and shown in Fig. 1B. As QSI and AHL bind to LasR competitively, the inhibiting effect of QSI is less efficient. Irreversible QSIs like halogenated furanones^35^ that induce degradation of the LasR receptor protein can inhibit QS more effectively (simulation data not shown). When combined, QQE and QSI can enhance the inhibiting effects of each other (Fig. 1C and D). 0.5 µM QSI alone has very little effect, but it can reduce the minimum QQE rate required to turn off QS up to four-fold. Similarly, adding a small amount of QQE (${\rm{\eta }}({\rm{QQ}})=0.5\times {10}^{-4}{s}^{-1}$) can reduce the minimum QSI concentration required to turn off QS up to 20-fold. The stationary AHL concentration decreased as compared to single treatment using QSI.

**Figure 2:**
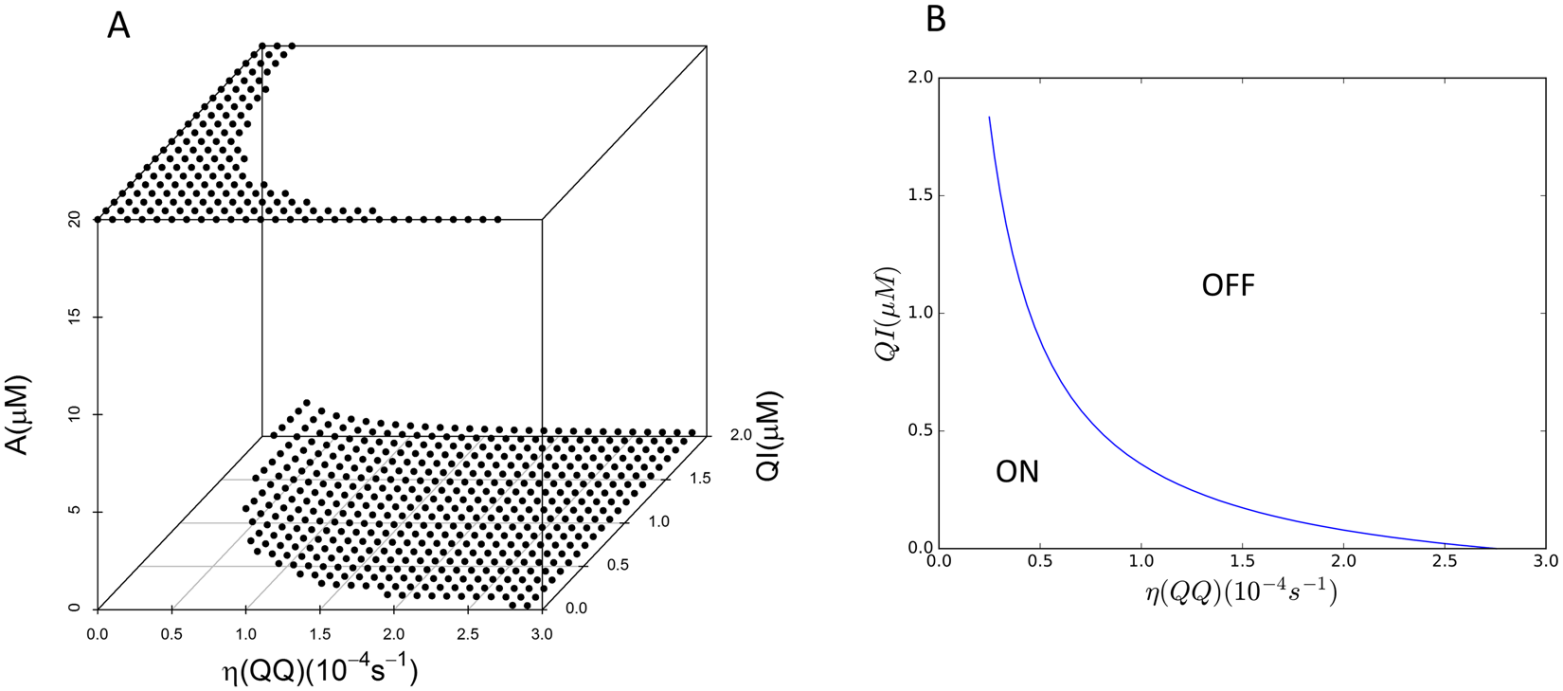
Simulation QS states to QQ and QSI. (**A**) 3D stationary AHL concentration to η(QQ) and QSI, (**B**) 2D map of QS on and off states to η(QQ) and QSI.

3D plot of stationary AHL to *η*(QQ) and QSI is shown in Fig. 2A. A clear boundary between QS on and off states was observed, which is shown in Fig. 2B. This boundary curve is “U”-shaped which means QQE and QSI have a synergistic effect in inhibiting QS^36^. *η*(QQ) is assumed to be proportional to QQE in this simulation. However, if other enzymatic dynamics such as the Michaelis–Menten equation^37^ was used, ${\rm{\eta }}^{\prime} ({\rm{QQ}}) > 0$ and ${\rm{\eta }}^{\prime\prime} ({\rm{QQ}})\le 0$ are satisfied. If we change the *η*(QQ) axis to QQE in Fig. 2B, the curve will still be “U”-shaped and the conclusions of the simulation will remain the same.

### Combination therapy of QSI and QQE inhibits *las* and *pqs* expression of *P. aeruginosa* bioreporter strains

**Figure 3:**
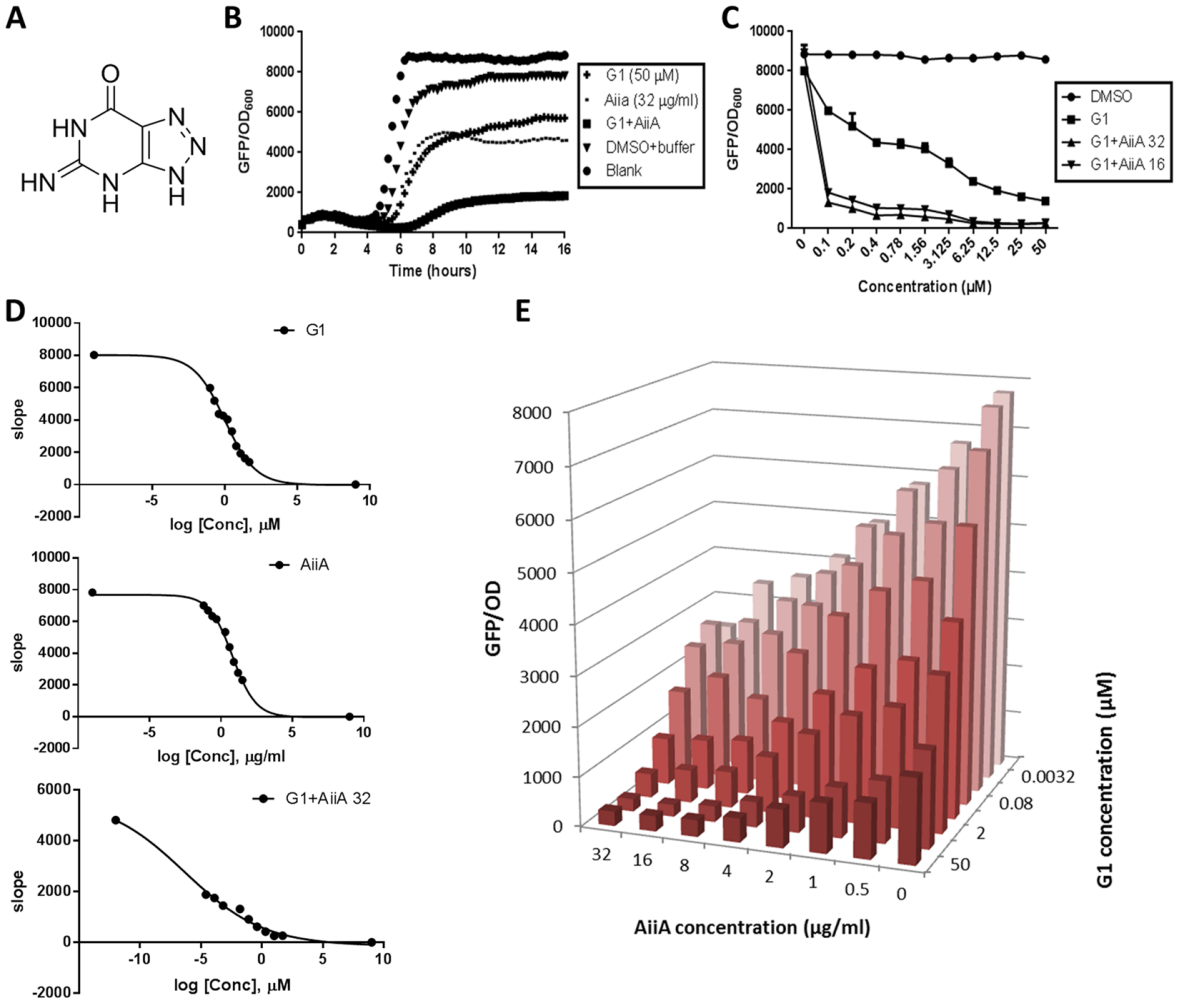
(**A**) Structure of G1. (**B**) Combination effects of G1 and AiiA on PAO1-*lasB-gfp* reporter strain. (**C**) Dose-response effects of G1 (50 µM) added with AiiA (32 µg/ml and 16 µg/ml) on *lasB-gfp*. (**D**) IC_50_ values of the single and combination treatments on *lasB-gfp*, G1 (1.36 ± 0.08 µM), AiiA (6.88 ± 0.46 µg/ml), G + A 32 µg/ml (4.45 ± 0.15 nM). Calculation was performed using GraphPad Prism 6 software, taken at the time point between 4–6 hours where the inhibition started to occur. (**E**) Dose-pair effects of G1 and AiiA on *lasB-gfp*. The concentration of DMSO and buffer correspond to the highest test concentration used in the assays. All experiments were done in triplicate manner, only representative data are shown.

To validate the mathematical modeling results, the combination effects of QSI and QQE were tested using *P. aeruginosa* QS bioreporter strain PAO1-*lasB-gfp*^38^. In our study, we chose AiiA as QQE in combination with G1, a small molecule of QSI which binds to LasR and RhlR^30^ as our models (Fig. 3A). The elastase encoding *lasB* gene is controlled by LasR^39^, hence any reduction in the fluorescence signals would indicate the presence of 3-oxo-C12-HSL antagonist. Both G1 and AiiA were tested at different concentration gradients to generate the dose-dependent curves, which are used to calculate the IC_50_ values. Most importantly, in support of our non-growth inhibitory antimicrobial principle^1^, neither G1 nor AiiA affected the growth rate of the bacteria (Supplementary Figure S2). The growth measured as OD_600_ was used as control of our non-growth inhibitory concept.

Combination effects of G1 and AiiA on *lasB-gfp* expression are summarized in Fig. 3. The effects were also observed in dose-dependent manner (Fig. 3C, Supporting Information Fig. S3). Both G1 and AiiA inhibited *lasB-gfp* expression in dose-dependent manner with IC_50_ values of 1.36 ± 0.08 µM and 6.88 ± 0.46 µg/ml respectively. Promising results were obtained in combinational therapy of AiiA and G1, where the IC_50_ values were significantly reduced to nanomolar range. The IC_50_ value calculated for G1 when combined with 32 µg/mL of AiiA was 4.45 ± 0.15 nM (Fig. 3D).

Next, we investigated if the combination therapy would also affect the PQS system: the third intercellular signaling mechanism of *P. aeruginosa* that regulates numerous virulence factors, including those involved in iron scavenging and apoptosis of host cells^40,41^. PQS is under positive regulation of LasR and negative regulation of RhlR^40,42^. PQS has also been detected in the lungs of cystic fibrosis patients^43^ and reported to suppress host innate immune responses through nuclear factor-κB pathway^44^.

**Figure 4:**
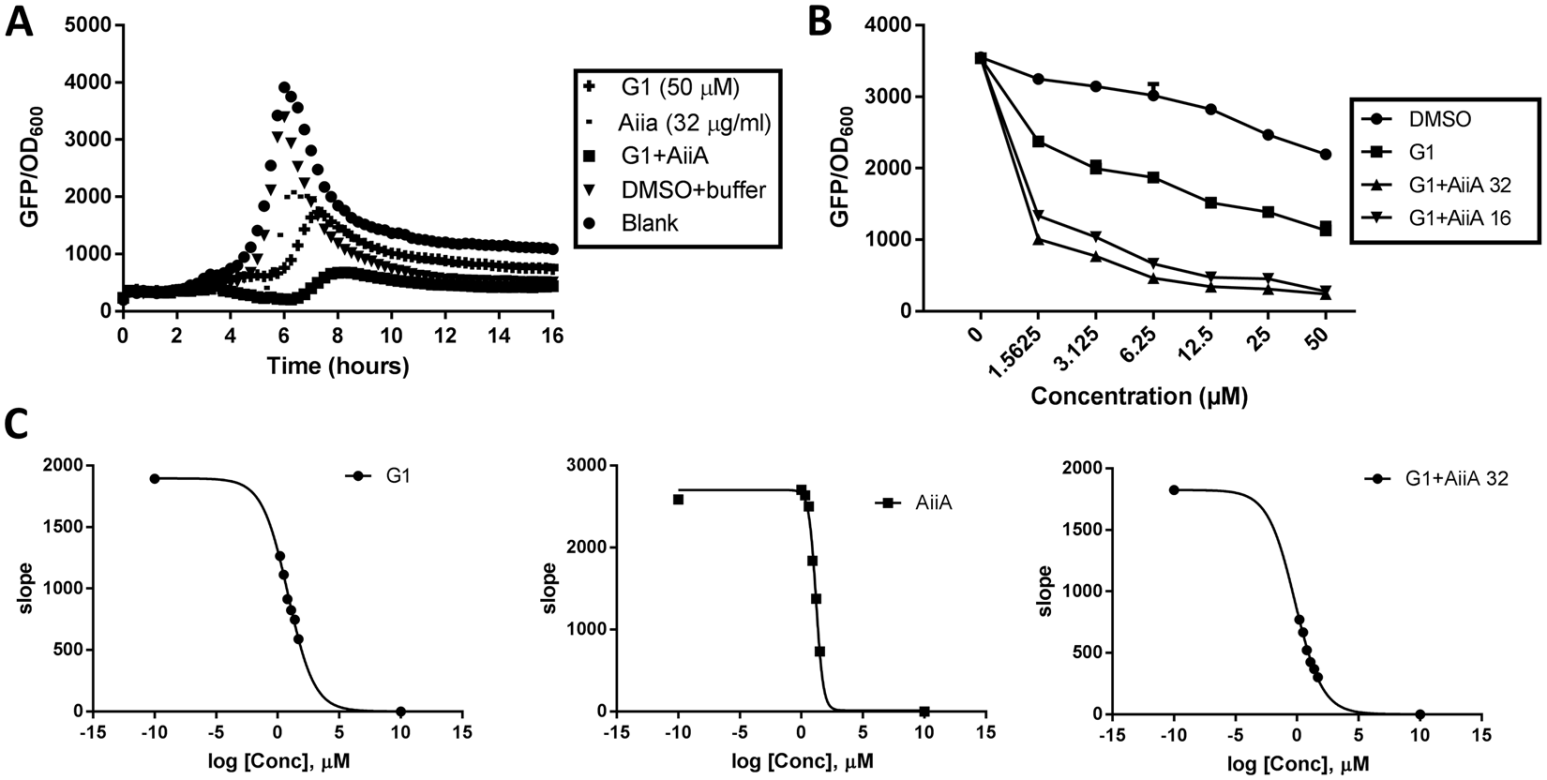
(**A**) Combination effects of G1 and AiiA on PAO1-*pqsA-gfp* reporter strain. (**B**) Dose-response effects of G1 (50 µM) added with AiiA (32 µg/ml and 16 µg/ml) on *pqsA-gfp*. (**C**) IC_50_ values of the single and combination treatments on *pqsA-gfp*, G1 (3.08 ± 0.72 µM), AiiA (15.58 ± 0.17 µg/ml), G + A 32 µg/ml (0.63 ± 0.06 µM). Calculation was performed using GraphPad Prism 6 software, taken at the time point between 4–6 hours where the inhibition started to occur. Experiments were done in triplicate manner, only representative data are shown.

For this experiment, we tested the compounds against *pqsA-gfp* reporter strain. The biosynthesis of PQS and other classes of alkyl quinolones requires genes encoded by the *pqsABCDE* and *phnAB* operons^45^. Combination treatment reduced *pqsA-gfp* expression, and the effects were also observed in a dose-dependent manner (Fig. 4A and B, Supporting Information Fig. S4). The IC_50_ value of combined treatment was reduced up to five-fold as compared to single treatments (Fig. 4C). To confirm the significance of our data, 2-way ANOVA analysis was performed and multiple comparisons test indicated significant p values across various combination doses for different reporter strains (Suppporting Information Table 2).

### G1 could not inhibit both *las* and *rhl* QS systems alone

We next examined the effects of QSI on the *rhl* QS system, which regulates many QS-dependent virulence factors once activated upon formation of RhlR-C4-HSL^10,46^. The AiiA has been experimentally shown to degrade C4-HSL^47^. Our previous experiments also showed that G1 was able to inhibit *rhl* system more effectively in *P. aeruginosa lasR* mutant but not the *rhl* system in the PAO1 wildtype^30^. We thus hypothesized that G1 has a different binding affinity to LasR than RhlR in the PAO1 wildtype and its intracellular concentration is not high enough to repress both LasR and RhlR simultaneously.

**Figure 5:**
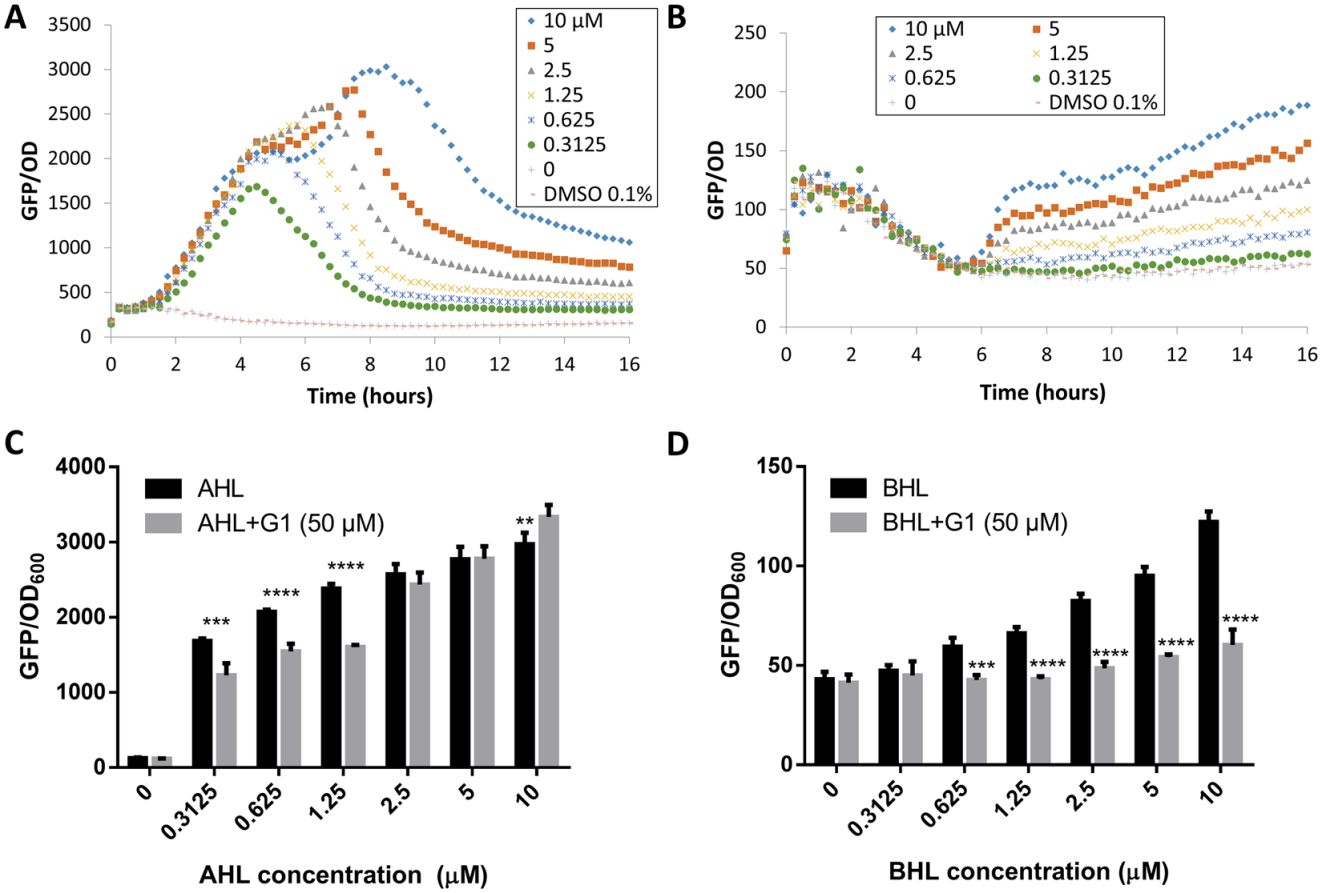
(**A**) Dose-dependent curves of QS deficient Δ*lasI*Δ*rhlI* double mutant harboring *lasB-gfp* supplemented with 3-oxo-C12-HSL (AHL). (**B**) Dose-dependent curves of QS deficient Δ*lasI*Δ*rhlI* double mutant harboring *rhlA-gfp* supplemented with C4-HSL (BHL). (**C**) Expression of the Δ*lasI*Δ*rhlI* double mutant harboring *lasB-gfp* when G1 was added together with AHL. (**D**) Expression of the Δ*lasI*Δ*rhlI* double mutant harboring *rhlA-gfp* when G1 was added together with BHL. Experiments were done in triplicate manner, only representative data are shown. Error bars are means ± SDs. ** = p < 0.01, *** = p < 0.001, **** = p < 0.0001, two-way ANOVA test.

To test this hypothesis, we examined the competitive binding efficacy of G1 with 3-oxo-C12-HSL and C4-HSL using a QS deficient *P. aeruginosa* Δ*lasI*Δ*rhlI* double mutant which can respond to the addition of exogenous AHLs (3-oxo-C12-HSL and C4-HSL respectively). In this setting, only one QS system is activated at one time. The reporter strains showed dose-dependent curves when supplemented with a different concentration of 3-oxo-C12-HSL and C4-HSL (Fig. 5A and B). Next, using the same setting, 50 µM of G1 was added together with the exogenous AHLs and the expression profiles were monitored for up to 16 hours (Supporting Information Fig. S5).

When 50 µM of G1 was added together with 3-oxo-C12-HSL, reduction in *lasB-gfp* was only observed when the concentration of 3-oxo-C12-HSL is less than 2.5 µM (Fig. 5C). However, G1 was able to reduce *rhlA-gfp* expression with all tested C4-HSL concentrations up to the basal level (Fig. 5D). Comparing our data with previously reported findings^30^, we proposed that G1 has a higher affinity to RhlR than LasR. However because most of the intracellular G1 was ‘consumed’ due to LasR abundance, hence its effect to inhibit *rhl* QS in the PAO1 wildtype was abolished due to earlier induction of *las* QS than the *rhl* QS during growth.

### AiiA enhances inhibition of G1 on *rhl* QS system in *P. aeruginosa*

Since we observed strong inhibition effects on *las* system, we hypothesized that our combination therapy strategy could also be applied to the *rhl* system. AiiA could act extracellularly and degrade 3-oxo-C12-HSL, therefore less amount of 3-oxo-C12-HSL would enter the cell and activate the LasR production. Low abundance of LasR protein would ‘consume’ less amounts of G1 hence more of these molecules could bind with RhlR.

**Figure 6:**
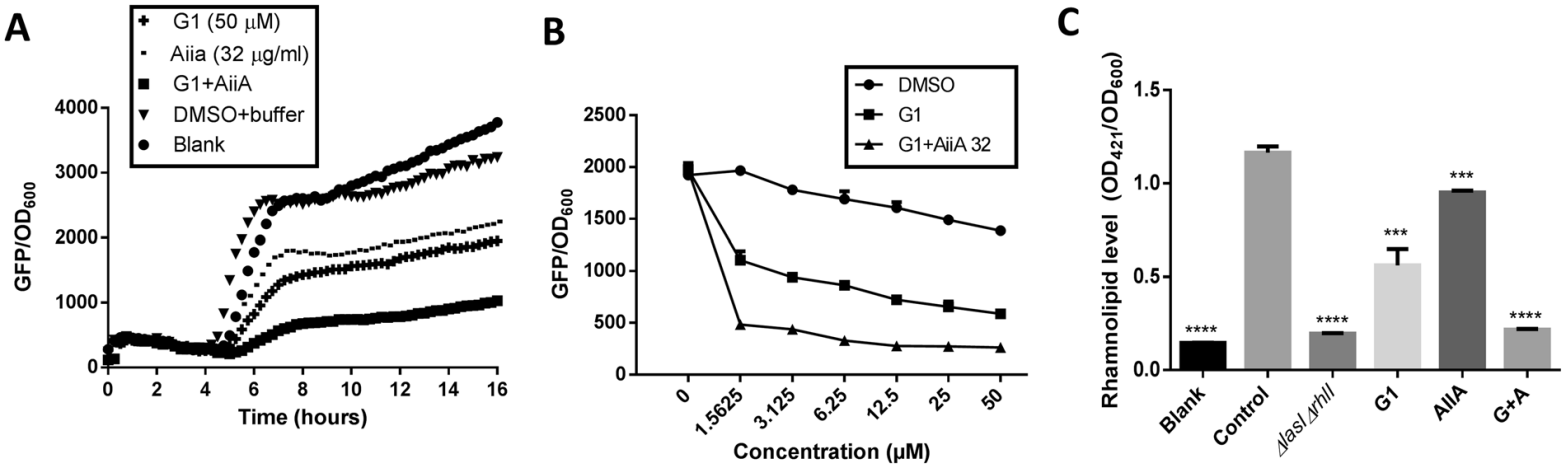
Effects of QQ and QSI on the *rhl* system. (**A**) Combination effects of G1 and AiiA on PAO1-*rhlA-gfp* reporter strain. (**B**) Dose-response effects of G1 (50 µM) added with AiiA (32 µg/ml) on *rhlA-gfp*. (**C**) Effects on rhamnolipid production when tested at final concentration of 50 µM (for G1), and 32 µg/mL (for AiiA). Same amount of DMSO and buffer were used as positive control. PAO1 Δ*lasI*Δ*rhlI* was used as negative control. Experiments were done in triplicate manner. Error bars are means ± SDs. *** = p < 0.001. **** = p < 0.0001, Student’s t test.

To test our hypothesis, we used PAO1-*rhlA-gfp* bioreporter strain to evaluate the combination effects. The *rhlA* is the first gene of the *rhlAB* operon that codes for the rhamnolipid biosynthesis^46^. We observed similar findings where the *rhlA-gfp* activity was highly suppressed in the combination treatment (Fig. 6A). The effects were also observed in dose-dependent manner (Fig. 6B, Supporting Information Fig. S6). IC_50_ value calculated for the combination treatment between G1 and AiiA for the *rhlA-gfp* expression is 1.38 ± 0.16 µM, much lower than the single treatments (IC_50_ for G1 = 3.65 ± 0.95 µM and AiiA = 17.79 ± 1.77 µg/ml, Supporting Information Fig. S7).

Next, we were interested to investigate if the combination effects could reduce the virulence of *P. aeruginosa*. We decided to test the rhamnolipid production, as it is one of the key QS-regulated virulent factors in the early stages of infection. Rhamnolipid promotes infiltration of respiratory epithelial cells^48^ and promote rapid necrotic killing of polymorphonuclear (PMNs) leukocytes^49^. Rhamnolipid is also critical in each stage of biofilm formation and contribute to the structure of biofilms^50,51^.

In the rhamnolipid assay, overnight culture of PAO1 was adjusted to OD_600_ 0.01 and grown in the presence of AiiA, G1 and combination of AiiA and G1 for 18 hours. The rhamnolipid was then extracted and quantified using the orcinol assay^52^. Treatment with AiiA alone did not fully decrease rhamnolipid production. However, when combined with G1, the rhamnolipid production was almost diminished to a similar level to the QS-defective Δ*lasI*Δ*rhlI* mutant (Fig. 6C). The findings correlate well with the results obtained from inhibition of *rhlA-gfp* bioreporter strain. The experimental results showed promising application of QQE and QSI in reducing virulence factors associated with host infection.

## Discussion

Over the years, the emergence of multidrug-resistant bacteria and shortage of new antibiotics have been seen as critical issues and the greatest threat to human health. The anti-virulence approach has been long considered as an alternative in controlling the pathogenicity and reducing resistance development^1^. Bacterial QS systems have been proposed as ideal targets for the development of anti-virulence drugs. However, no QS interference compounds have been applied clinically till date. Many of the QSIs are developed via targeting the AHL-mediated QS systems. The co-existence of multiple AHL QS systems in individual bacterial species might thus be a challenge for the application of QSIs in clinical settings.

In this study, we used mathematical modeling of *P. aeruginosa* LasR/I QS network in batch culture to show that combination therapy of QQE and QSI has combinatorial effects in suppressing the *las* QS. Simulation results show that very large *η*(QQ) or QSI concentration was needed to inhibit QS. Strong combination effect was observed between QQE and QSI. Interestingly, switching of QS circuit in the simulations was not observed in experiments. This might be due to simplification of the mathematical model, which assumes every cell is homogeneous and synchronized. In the single-cell study of QS signaling in *V. fischeri*, switching behaviour was observed while tracking single cells but the population level fluorescence was a graded response^33^. This could also be similar to the case of the lactose utilization network, where switching was observed at the single cell but not at the population level^53^.

The experimental results showed promising application of QQE and QSI based on different bioreporter assays. The AHL-dependent QS system has been an attractive target to control bacterial pathogenicity as it controls the expression of a wide range of virulence genes. The AiiA enzyme has been reported to show high specificity and preference towards different signal molecules (acyl chain length and substitution)^47^ and demonstrated to reduce the concentration of 3-oxo-C12-HSL based on our HPLC analysis (Supplementary Figure S8). In some cases, the degradation of QS signal alone is not sufficient to completely diminish and block the QS activities^29^. AiiA could abolish and effectively quench the AHL signal molecules, however it was surprising to see its much lesser effect on the PQS system, which was also under *las* regulation. Combination treatment with G1 resulted in significant reduction of *lasB, pqsA*, and *rhlA-gfp* expression as compared to single treatment of both AiiA and G1. Our work demonstrated that combining two classes of QSI and QQE could provide multiple points of attacks and efficient blockade of QS-mediated signaling pathways.

In our previous study, G1 has been shown to interact and compete with AHL to inhibit LasR in *P. aeruginosa*^30^. In *P. aeruginosa* PAO1 strain, where both *las* and *rhl* circuits exist, G1 could only inhibit *las* system but not *rhl* system. However, when *lasR* was mutated, G1 could effectively inhibit *rhl* system. In our competitive binding assay using bioreporter strains, we demonstrated that QSIs might have different affinities to QS receptor proteins. G1 could inhibit *lasB-gfp* expression only when concentration of 3-oxo-C12-HSL was smaller than 1.25 µM. However, the inhibition effect of G1 to *rhlA-gfp* was still significant even when the concentration of C4-HSL was 10 µM. We also observed the transcription rates of *las* system was activated first before *rhl*. The results explain why despite of its higher binding affinity to RhlR, G1 would still bind to LasR as the *las* system is activated first in PAO1. But in the case of *lasR* mutant, G1 has higher competitiveness to C4-BHL and could inhibit *rhl* system effectively.

The results also suggested why AiiA could enhance the inhibition effects of G1 on *rhl* system, as shown by our experimental data. Assuming intracellular concentration of G1 is constant, G1 would have to competitively bind to both LasR and RhlR. In this case, addition of AiiA to quench both 3-oxo-C12-HSL and C4-HSL resulted in lower abundance of LasR and therefore G1 could specifically bind to inhibit RhlR. Thus combination of QQE and QSIs might not only enhance the efficacy of QSIs, but also expand the targeting systems of QSIs as many bacteria have more than one *lux* QS systems.

Although LasR regulator has long been considered essential for full virulence of *P. aeruginosa*^54^, loss-of-function *lasR* mutants occur frequently in the natural environment^55^, individuals suffering from pneumonia, wound infections^56^ and in cystic fibrosis patients^57^. In the *lasR* mutants, the QS-regulated virulence factors continue to be expressed. There has also been reports that the *rhl* system could override the hierarchy of QS network in a non-functional *las* system^58^. Recent studies also showed that RhlR plays critical roles as QS regulator using *Drosophila melanogaster* oral infection model^59^ and controls pathogenesis and biofilm development^60^. This highlights the importance to develop new strategies that target multiple QS pathways, instead of a specific QS system to control pathogenicity. This could be achieved in two ways; firstly through exploitation of natural or synthetic compounds that have broad targets on QS pathways. The synthetic disulfide-containing ajoene analogs are examples of such class of compounds demonstrated to inhibit QS in *P. aeruginosa*^61^. Secondly, we propose new concept of combining two classes of compounds with different mechanism of actions in suppressing multiple QS pathways.

In conclusion, the combination effects of QQE and QSI compound have been demonstrated *in vitro* in this study. Mathematical modeling showed enhanced QS inhibiting effects on AHL concentration when QQE (AiiA) and QSI (G1) were applied together. We have also provided better understanding and elucidated QS network interaction with G1 in this work. The implication of our study represents a novel approach of utilizing QS-interfering compounds to impede virulence and block pathogenesis. Future work will aim to evaluate the effectiveness of the combined treatments *in vivo*.

## Materials and Methods

### General information

All chemicals were purchased from Sigma Aldrich and used without further purification. G1 (5-imino-4,6-dihydro-3H-1,2,3-triazolo[5,4-d]pyrimidin-7-one) was purchased from TimTec LLC (Newark, DE). Bacteria were grown in Luria-Bertani (LB) broth (1% tryptone, 0.5% yeast extract, and 0.5% NaCl). Media used for biological assay was ABTGC (AB minimal medium supplemented with 0.2% glucose and 0.2% casamino acids)^61^. Bacterial strains used in this study are shown in Table 1.

**Table 1:** Bacterial strains used in this study.

Strains	Relevant genotype and/or characteristics
PAO1	*Pseudomonas aeruginosa* wild type^12^
PAO1-*gfp*	GFP-tagged PAO1^65^
PAO1-*lasB-gfp*	PAO1 containing *lasB-gfp*(ASV) reporter fusion^12^
PAO1-*pqsA-gfp*	PAO1 containing *pqsA-gfp*(ASV) reporter fusion^65^
PAO1-*rhlA-gfp*	PAO1 containing *rhlA-gfp*(ASV) reporter fusion^65^
PAO1 Δ*lasI*Δ*rhlI*	PAO1 QS deficient *lasI and rhlI* double mutant^66^
PAO1 Δ*lasI*Δ*rhlI-lasB-gfp*	PAO1 *lasI and rhlI* mutant containing *lasB-gfp*(ASV) reporter fusion^67^
PAO1 Δ*lasI*Δ*rhlI -rhlA-gfp*	PAO1 *lasI and rhlI* mutant containing *rhlA-gfp*(ASV) reporter fusion^67^

### Expression and purification of QQ enzyme AiiA

The gene coding for AiiA was cloned into pET-47b(+) vector (BamHI-HindIII sites). The expression vector was transformed into *E. coli* BL21(DE3) competent cells (New England Biolabs, USA). The cells were grown in 2 L of LB media supplied with 35 mg/L Kanamycin at 37 °C. The expression of the protein was induced with 0.5 mM of Isopropyl β-D-1-thiogalactopyranoside (IPTG) when OD_600_ reached 0.6. The cells were grown overnight at 18 °C. The harvested cells were resuspended in 50 mL of lysis buffer (50 mM Tris-HCl, pH 8.0, 150 mM NaCl, 0.05% (v/v) CHAPS, 10% (v/v) glycerol) and lysed by passing the homogenized cells through an Emulsiflex-C3 (Avestin, USA) high-pressure apparatus at 15,000 psi three times. The cell lysate was centrifuged at 25,000 g for 25 min. The supernatant was then applied to Ni-NTA gravity column (Bio-rad) equilibrated with lysis buffer. After extensive washing with lysis buffer, the bound proteins were eluted with lysis buffer containing increasing concentration of imidazole (0–300 mM). The eluted fractions were analyzed with 15% of SDS-PAGE and fractions containing the desired protein were pooled and dialyzed against lysis buffer. The final concentration of the protein was measured using Bradford Assay.

### Enzymatic assay of AiiA

The 3-oxo-C12-HSL hydrolysis activity of AiiA was tested with 500 µM 3-oxo-C12-HSL, 30 µM AiiA in reaction buffer (20 mM Tris-HCl, 150 mM NaCl, pH 8.0). After 30 min of reaction at 30 °C, the reaction mixture was monitored at 215 nm by analytic C18 reverse phase HPLC column (Jupitar, 5 µ, 300 Å, 250 × 4.6 mm) with a flow rate of 0.5 mL/min (Gradient: 0–100% buffer B (90% acetonitrile, 10% H2O, 0.05% TFA) in buffer A (100% H2O, 0.05% TFA) for 50 min).

### Model of LasR/I circuit

The models in this study simulated batch cultures according to the experimental setup. The LasR/I QS circuit of *P. aeruginosa* is shown in Supplementary Figure S1. QSI binds to LasR similarly as AHL, but in this case only AHL can stabilize the LasR^62^. Since our focus of the present study was whether QQ enzyme and QSI have synergistic effect in inhibiting QS, some complex features in the QS network were simplified to make the model easier to implement and reduce computational cost. For instance, the interactions of LasR/I QS circuits with other cellular components, such as the binding of 3-oxo-C12-HSL to RhlR^21^ were not included in the network studied in this work. Both heterogeneity and asynchronization of cells were beyond the scope of the modeling in this work. The final component concentrations of cells in batch culture were adapted in the computation of AHL concentration. As the response time of QS switching is much faster than the time required for culture growth^33^, the final component concentrations can be approximated by the stationary concentrations with the final cell volume fraction **ρ**. The AHL concentration was considered to be homogeneous inside and outside cells due to its large diffusion coefficient^63^. Vfr was assumed to be some large enough constant since it is normally expressed in experimental strains of this study. QSI concentration was taken as a constant considering its relative big value. A maximum concentration of AHL **A**_**max**_ was set in the model to avoid very large concentrations of components caused by the accumulation of stable AHL in batch cultures^64^. The reactions of QS network are shown in Table 2. QQ enzyme and QSI are written as **Q**_**Q**_ and **Q**_**I**_ to avoid confusion when necessary.

**Table 2:** Biochemical reactions in *P. aeruginosa* LasR/I circuit.

Reaction	Description	Rate
I → I+A	Production of A	$\frac{{{\rm{V}}}_{{\rm{A}}}I}{{K}_{A}+I}$
A → null	Natural decay of A	${{\rm{d}}}_{{\rm{A}}}A$
R + A → P	Combination of R and A	${{\rm{k}}}_{{\rm{RA}}}RA$
P → R + A	Dissociation of P	${{\rm{d}}}_{{\rm{P}}}{\rm{P}}$
R → null	Decay of LasR protein	${{\rm{d}}}_{{\rm{R}}}R$
r → r + R	Translation of lasR mRNA	${{\rm{k}}}_{{\rm{r}}}r$
null → r	Transcription of lasR	${{\rm{r}}}_{0}+{V}_{r}Z(\frac{1-{e}^{-\beta V}}{{K}_{r1}+Z}+\frac{{e}^{-\beta V}}{{K}_{r2}+Z})$
r → null	Decay of lasR mRNA	${{\rm{d}}}_{{\rm{r}}}r$
${\rm{i}}\to {\rm{i}}+{\rm{I}}$	Translation of lasR mRNA	${{\rm{k}}}_{{\rm{i}}}i$
I → null	Decay of LasI protein	${{\rm{d}}}_{{\rm{I}}}I$
null → i	Transcription of lasI	${{\rm{i}}}_{0}+\frac{{V}_{i}Z}{{K}_{i}+Z}$
i → null	Decay of lasI mRNA	${{\rm{d}}}_{{\rm{i}}}i$
2P → Z	Dimerization of P	${{\rm{k}}}_{{\rm{Z}}}{P}^{2}$
Z → 2P	Dissociation of Z	${{\rm{d}}}_{{\rm{Z}}}Z$
${\rm{R}}+{{\rm{Q}}}_{{\rm{I}}}\to {\rm{F}}$	Combination of R and QSI	${{\rm{k}}}_{{\rm{RQ}}}R{Q}_{I}$
F → R + Q_I_	Dissociation of F	${{\rm{d}}}_{{\rm{F}}}F$
F → Q	Degradation of R in F	${{\rm{d}}}_{{\rm{R}}}F$
A → null	Degradation of A by QQ	${\rm{\eta }}({{\rm{Q}}}_{{\rm{Q}}})A$

The ordinary differential equations of the QS network are listed in equations 1 to 8. Stationary QS components were solved using the steady state condition. When there are multiple stable stationary solutions, the state with smallest concentrations was chosen as the outcome presented in this work. The parameters of *P*. *aeruginosa* LasR/I QS circuit were firstly estimated from reported values in the literature, then optimized to enable the switching behaviour of the QS network observed in experiments (Supplementary Table S1)^33,34^.

1 $$\frac{{\rm{dA}}}{{\rm{dt}}}={\rm{\rho }}\frac{{V}_{A}I}{{K}_{A}+I}+\rho {d}_{P}P-\rho {k}_{RA}RA-{d}_{A}A-(1-\rho )\eta ({Q}_{Q})A$$

2 $$\frac{{\rm{dR}}}{{\rm{dt}}}={k}_{r}r+{d}_{P}P-{k}_{RA}RA-{d}_{R}R+{d}_{F}F-{k}_{RQ}R{Q}_{I}$$

3 $$\frac{{\rm{dF}}}{{\rm{dt}}}={k}_{RQ}R{Q}_{I}-{d}_{F}F-{d}_{R}F$$

4 $$\frac{{\rm{dr}}}{{\rm{dt}}}=\frac{{r}_{0}}{{V}_{b}}+\frac{{V}_{r}Z}{{V}_{b}}(\frac{1-{e}^{-\beta V}}{{K}_{r1}+Z}+\frac{{e}^{-\beta V}}{{K}_{r2}+Z})-{d}_{r}r$$

5 $$\frac{{\rm{dP}}}{{\rm{dt}}}={k}_{RA}RA-{d}_{P}P+2{d}_{Z}Z-2{k}_{Z}{P}^{2}$$

6 $$\frac{dZ}{dt}={{\rm{k}}}_{{\rm{Z}}}{P}^{2}-{d}_{Z}Z$$

7 $$\frac{di}{dt}=\frac{{{\rm{i}}}_{0}}{{V}_{b}}+\frac{{V}_{i}}{{V}_{b}}\frac{Z}{{K}_{i}+Z}-{d}_{i}{\rm{i}}$$

8 $$\frac{{\rm{dI}}}{{\rm{dt}}}={k}_{i}i-{d}_{I}I$$

### Reporter gene assay

Stock solution of G1 was prepared by dissolving appropriate amount of chemicals in DMSO to make final concentration of 10 mM, aliquoted into small Appendorf tube and stored at −20 °C until further usage. The compound was then dissolved in ABTGC medium to the working concentration and 100 µL of this solution was pipetted into first rows of 96-well microtiter dish (Nunc, Denmark). Two-fold serial dilution was made to the rest of the rows, leaving the last two rows empty for blank and solvent control. Next, 50 µL of AiiA diluted in ABTGC media was added into each well. Overnight culture of *P. aeruginosa* reporter strain PAO1-*lasB-gfp* was diluted to optical density at 600 nm (OD_600_) of 0.02 (approximately 2.5 × 10^8^ CFU/mL). 100 µL of the bacterial suspension was added to each well and the plate was incubated for at least 16 hours at 37 °C. GFP fluorescence (excitation at 485 nm, emission at 535 nm) and OD_600_ readings were recorded every 15 mins using Tecan Infinite 200 Pro plate reader (Tecan Group Ltd, Männedorf, Switzerland). For the IC_50_ value calculation, it was determined at the time point where inhibition started to occur (between 4–6 hours) using Graphpad Prism 6 software. All assays were done in triplicate manner.

### Rhamnolipid quantification

Rhamnolipid was extracted and quantified using method reported by Koch *et al*. with modifications^52^. Briefly, overnight culture of *P. aeruginosa* was diluted to OD_600_ 0.01 in ABTGC medium. Into the cultures, compounds were added to appropriate concentration and the cultures were grown for 18 h at 37 °C (shaking at 200 rpm). Supernatants were collected and extracted with diethyl ether twice. The organic fractions were collected and concentrated to give white solids, which were further dissolved in water. 0.19% (w/v) orcinol in 50% H_2_SO_4_ was freshly prepared and added into the water solution. It was then heated at 80 °C for 20–30 min to give yellow-orange solution. The solution was allowed to cool at room temperature before measuring the absorbance at 421 nm. The results were normalized with cell density at OD_600_. Experiments were done in triplicate manner.

## Electronic supplementary material

Supporting Information file
