# Supplementary material for: Combination Therapy Strategy of Quorum Quenching Enzyme and Quorum Sensing Inhibitor in Suppressing Multiple Quorum Sensing Pathways of P. aeruginosa
Source: Sci Rep. 2018 Jan 18;8:1155. doi: 10.1038/s41598-018-19504-w (PMC5773576; doi:10.1038/s41598-018-19504-w)
Supplement: Supplementary file 1 — Supporting Information file [file 41598_2018_19504_MOESM1_ESM.pdf]

SUPPORTING INFORMATION

Combination Therapy Strategy of Quorum Quenching Enzyme and Quorum Sensing Inhibitor in Suppressing Multiple Quorum Sensing Pathways of *P. aeruginosa*

July Fong[1+], Chaodong Zhang[2+], Renliang Yang[1,3], Zhao Zhi Boo[1,3], Soon Keat Tan[4], Thomas E. Nielsen[5,6], Michael Givskov[5,6], Xue-Wei Liu[7], Bin Wu[1,3], Haibin Su[8*], Liang Yang[1,5*]

[1]School of Biological Sciences, Nanyang Technological University, 60 Nanyang Drive, Singapore 637551

[2]Nanyang Environment and Water Research Institute (NEWRI), Interdisciplinary Graduate School, Nanyang Technological University, 50 Nanyang Avenue, Singapore 639798

[3]NTU Institute of Structural Biology, Nanyang Technological University, EMB 06-01, 59 Nanyang Drive, Singapore 636921

[4]School of Civil and Environmental Engineering, Nanyang Technological University, 50 Nanyang Ave, Singapore 639798

[5]Singapore Centre for Environmental Life Sciences Engineering (SCELSE), Nanyang Technological University, 60 Nanyang Drive, Singapore 637551

[6]Costerton Biofilm Center, Department of Immunology and Microbiology, University of Copenhagen, 2200 København N, Denmark

[7]School of Physical & Mathematical Sciences, Nanyang Technological University, 21 Nanyang Link, Singapore 637371

[8]School of Materials Science and Engineering, Nanyang Technological University, 50 Nanyang Avenue, Singapore 639798

*Address correspondence to Haibin Su, HBSu@ntu.edu.sg and Liang Yang, yangliang@ntu.edu.sg.

+ J.F. and C.Z. contributed equally to this work.

Parameter	Description	Value	Unit	Reference
V_b	Average cell volume	1.37	μm^3	(1)
V_A	AHL max production rate	181.476	nM/s	(2, 3)
K_A	Half rate constant of AHL production	1585.6	nM	optimized
k_{RA}	Combination rate of LuxR and AHL	3.2×10^{-5}	$nM^{-1}s^{-1}$	(4, 5)
d_P	Dissociation rate of P	6.265×10^{-3}	s^{-1}	(4, 5)
r_0/V_b	Basal transcription rate of lasR	2.24×10^{-4}	nM/s	(4, 5)
i_0/V_b	Basal transcription rate of lasI	7.52×10^{-5}	nM/s	(4, 5)
d_r	Degradation rate of lasR mRNA	4.286×10^{-4}	s^{-1}	(3, 6)
d_i	Degradation rate of lasI mRNA	5.682×10^{-4}	s^{-1}	(3, 6)
V_r/V_b	Activated transcription rate of lasR	0.021	nM/s	(5)
V_i/V_b	Activated transcription rate of lasI	0.083	nM/s	(5)
K_{r1}	Half rate constant of lasR transcription with Vfr	4.125	nM	(5, 7)
K_{r2}	Half rate constant of lasR transcription without Vfr	50	nM	assumed
K_i	Half rate constant of lasI transcription	122.43	nM	(5)
k_r	Translational rate of lasR	7.794×10^{-3}	s^{-1}	(4, 5)
k_i	Translational rate of lasI	7.15×10^{-3}	s^{-1}	(4, 5)
d_R	Degradation rate of LasR	6.139×10^{-4}	s^{-1}	(4, 5, 8)
d_I	Degradation rate of LasI	8.294×10^{-4}	s^{-1}	(4, 5, 8)
d_A	Natural degradation rate of AHL	3.0×10^{-6}	s^{-1}	(9)
k_Z	Dimerization rate of P	2.803×10^{-6}	$nM^{-1}s^{-1}$	(4, 5)
d_Z	Dissociation rate of Z	0.0191	s^{-1}	(4, 5)
k_{RQ}	Combination rate of LasR and QSI	3.23×10^{-5}	$nM^{-1}s^{-1}$	use K_{RA}

d_F	Dissociation rate of F	6.265×10^{-3}	s^{-1}	use d_P
A_{max}	Max AHL concentration allowed in model	20	μM	assumed
ρ	Final cell volume fraction	0.3	1	estimated

Supplementary Table S1. Parameters used for modeling LasR/I circuit

Concentration (µM)	*lasB-gfp*			*pqsA-gfp*			*rhlA-gfp*	
	**DMSO vs G1**	**G1 vs G+A 32**	**G1 vs G+A 16**	**DMSO vs G1**	**G1 vs G+A 32**	**G1 vs G+A 16**	**DMSO vs G1**	**G1 vs G+A 32**
0	***	ns	**	ns	ns	ns	ns	ns
1.5625	****	****	****	****	****	****	****	****
3.125	****	****	****	****	****	****	****	****
6.25	****	****	****	****	****	****	****	****
12.5	****	****	****	****	****	****	****	****
25	****	****	****	****	****	****	****	****
50	****	****	***	****	****	****	****	****

Supplementary Table S2. Two-way ANOVA analysis of the dose-response effects between single and combination treatment of G1 (50 µM) and AiiA (32 µg/mL and 16 µg/mL) at QS reporter strains. Calculations were done using GraphPad Prism 6 software based on Bonferroni's multiple comparisons test. ns = $p > 0.05$, ** = $p < 0.01$, *** = $p < 0.001$, **** = $p \leq 0.0001$.

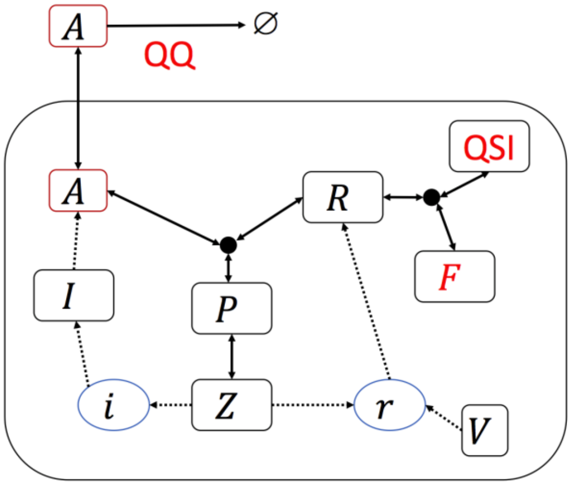

symbol	component
r	lasR mRNA
R	LasR protein
A	3-oxo-C12-HSL(AHL)
i	lasI mRNA
I	LasI protein
P	*R-A*
Z	dimer of *P*
V	Vfr
QSI	QSI
F	*R-QSI*

Supplementary Figure S1. *P.aeruginosa* LasR/I circuit model with QQ and QSI both indicated in red. Dashed lines indicate the reactants still remains after the reactions and solid lines indicate the reactants will disappear after the reactions.

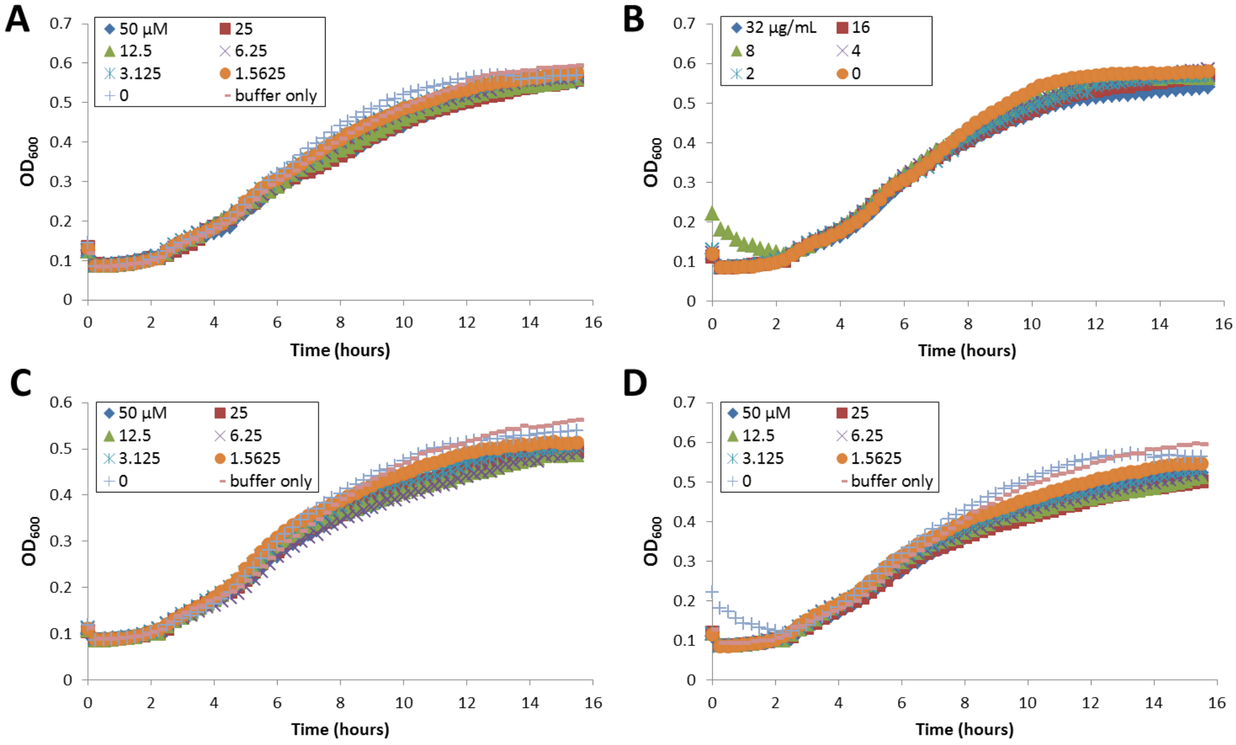

Supplementary Figure S2. Representative growth curves of *P. aeruginosa* incubated with compounds at different concentrations. Shown above are growth curves of (A) G1, (B) AiiA, (C) G1 and AiiA at 32 μg/mL, and (D) G1 and AiiA at 16 μg/mL respectively**.** The compounds didn't show any growth inhibition effect on *P. aeruginosa.* Growth curve was measured using Tecan Infinite 200 Pro plate reader (Tecan Group Ltd, Männedorf, Switzerland). Experiments were done in triplicate manner.

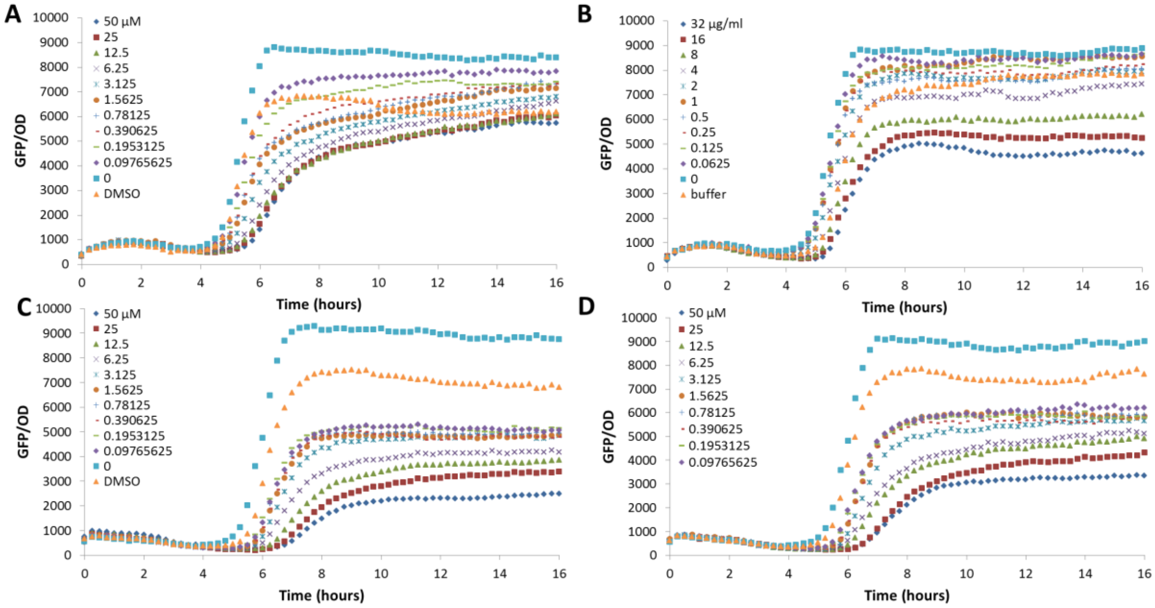

Supplementary Figure S3. Dose-dependent curves of compounds with QS reporter strain PAO1-*lasB*-*gfp*. (A) G1, (B) AiiA, (C) G1 and AiiA at 32 µg/mL, (D) G1 and AiiA at 16 µg/mL. Measurements were done using Tecan Infinite 200 Pro plate reader (Tecan Group Ltd, Männedorf, Switzerland). Experiments were done in triplicate manner.

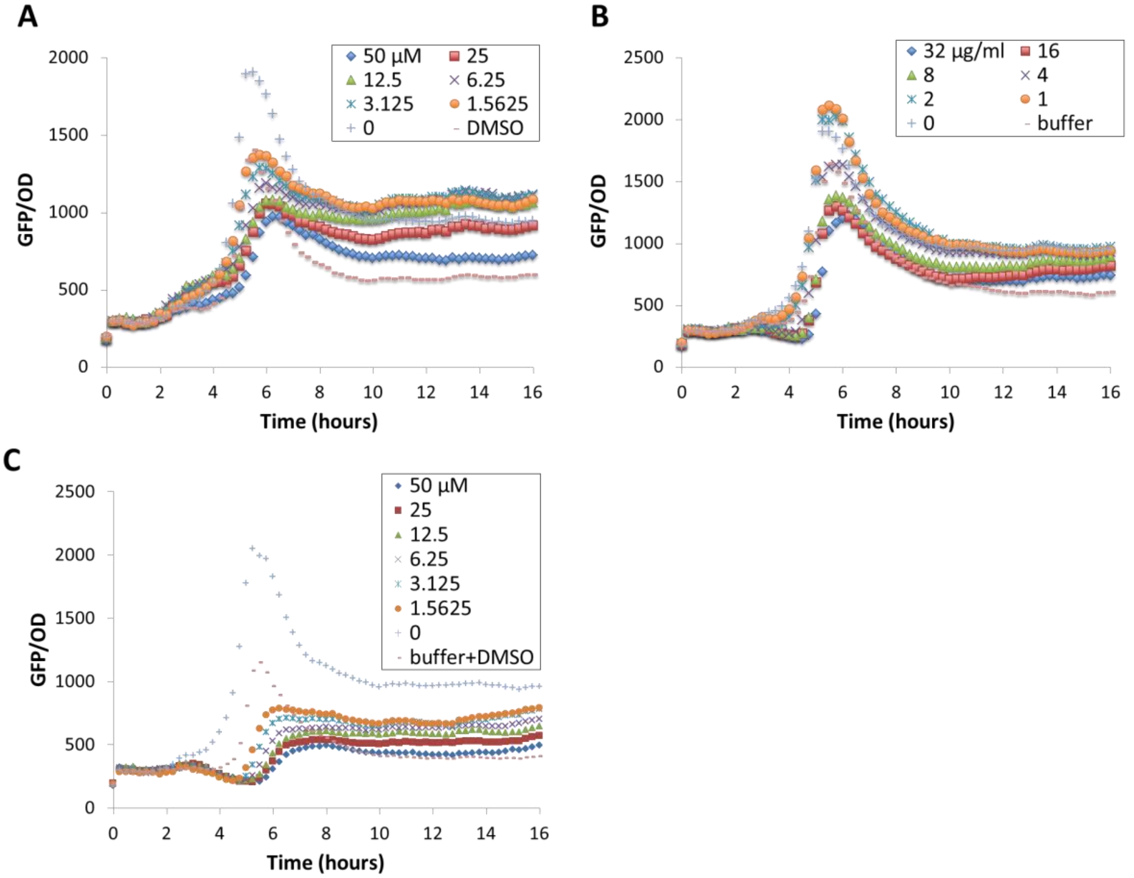

Supplementary Figure S4. Dose-dependent curves of compounds with QS reporter strain PAO1-*pqsA-gfp*. (A) G1, (B) AiiA, and (C) G1 and AiiA at 32 µg/mL. Measurements were done using Tecan Infinite 200 Pro plate reader (Tecan Group Ltd, Männedorf, Switzerland). Experiments were done in triplicate manner.

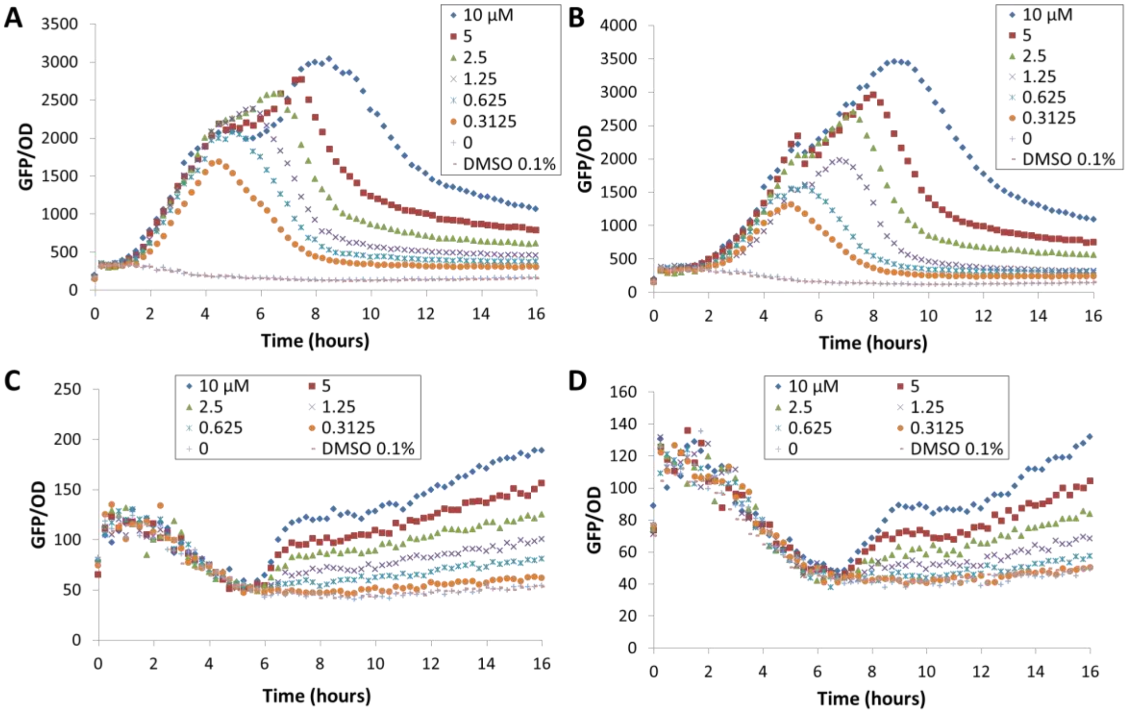

Supplementary Figure S5. Dose-dependent curves of QS deficient Δ*lasI*Δ*rhlI* double mutant harboring *lasB-gfp* (top) and *rhlA-gfp* (bottom) supplemented with (A) 3-oxo-C12-HSL, (B) 3-oxo-C12-HSL with G1 50 µM, (C) C4-HSL, and (D) C4-HSL with G1 50 µM. Experiments were done in triplicate manner, only representative data are shown.

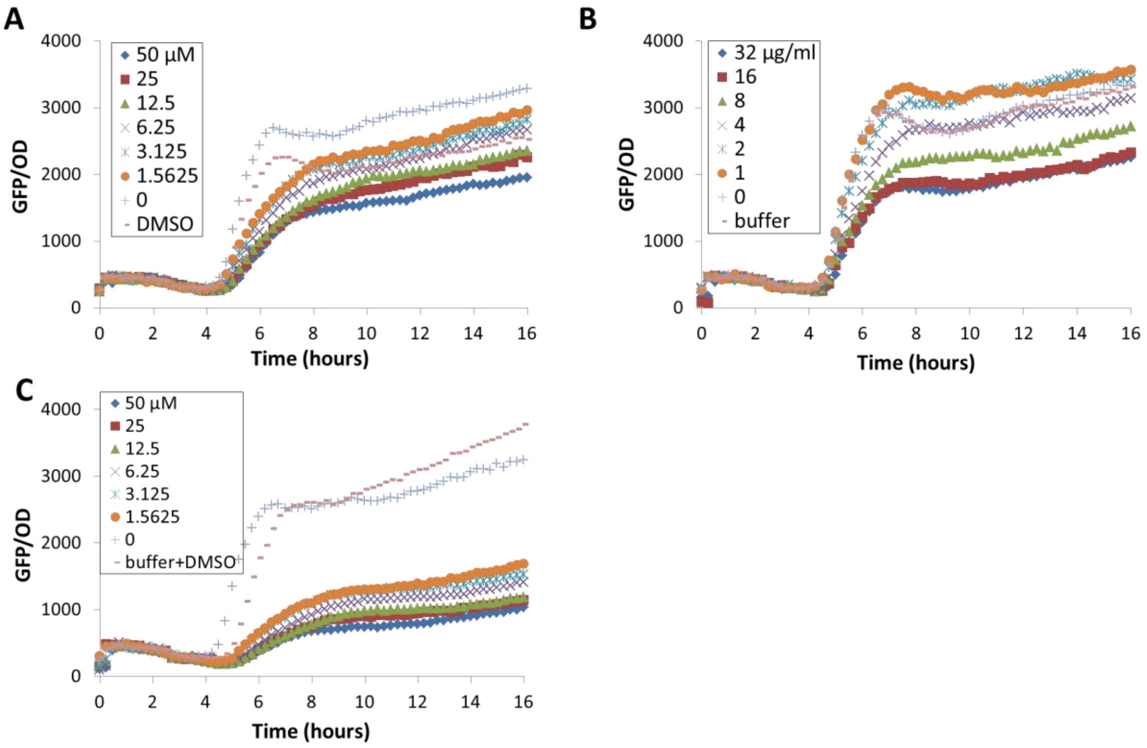

Supplementary Figure S6. Dose-dependent curves of compounds with QS reporter strain PAO1-*rhlA*-*gfp*. (A) G1, (B) AiiA, and (C) G1 and AiiA at 32 µg/mL. Measurements were done using Tecan Infinite 200 Pro plate reader (Tecan Group Ltd, Männedorf, Switzerland). Experiments were done in triplicate manner.

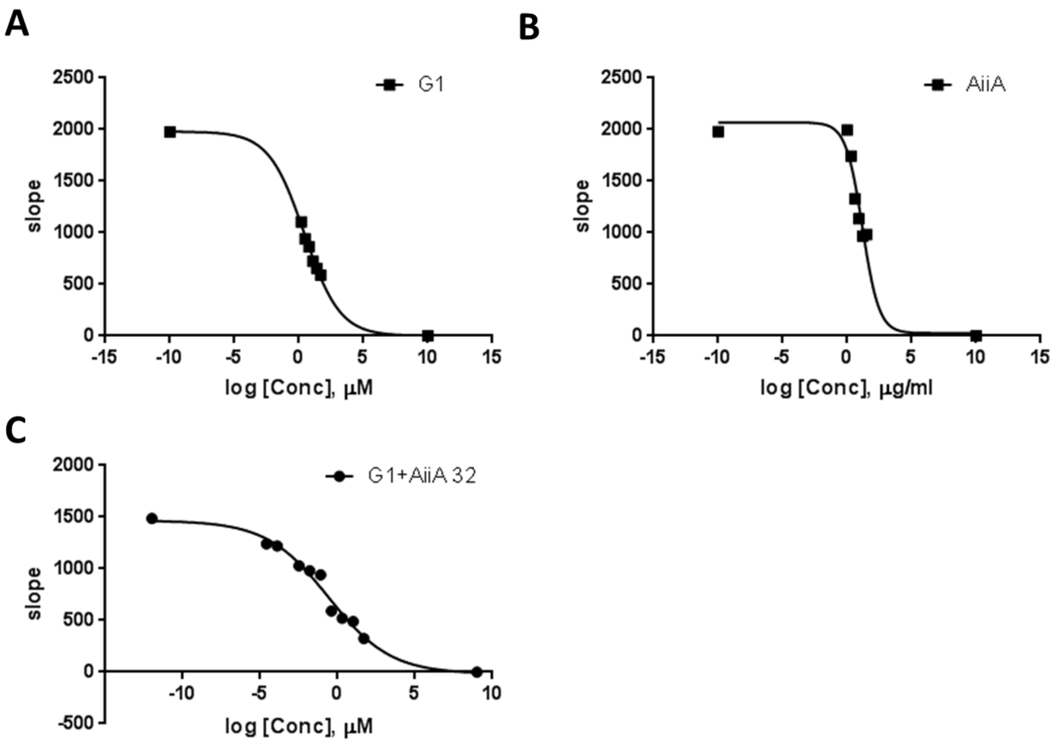

Supplementary Figure S7. IC_{50} values of compounds and combination therapy for PAO1-*rhlA*-*gfp*. For the IC_{50} values calculation, it was determined at the time point where inhibition started to occur (between 4-6 hours). Calculation are based on three biological replicates and done using GraphPad Prism 6 software. The IC_{50} values for G1is 3.65±0.95 µM and 17.79±1.77 µg/ml for AiiA. Addition of 32 µg/mL of AiiA to G1 reduced IC_{50} values to 1.38±0.16 µM.

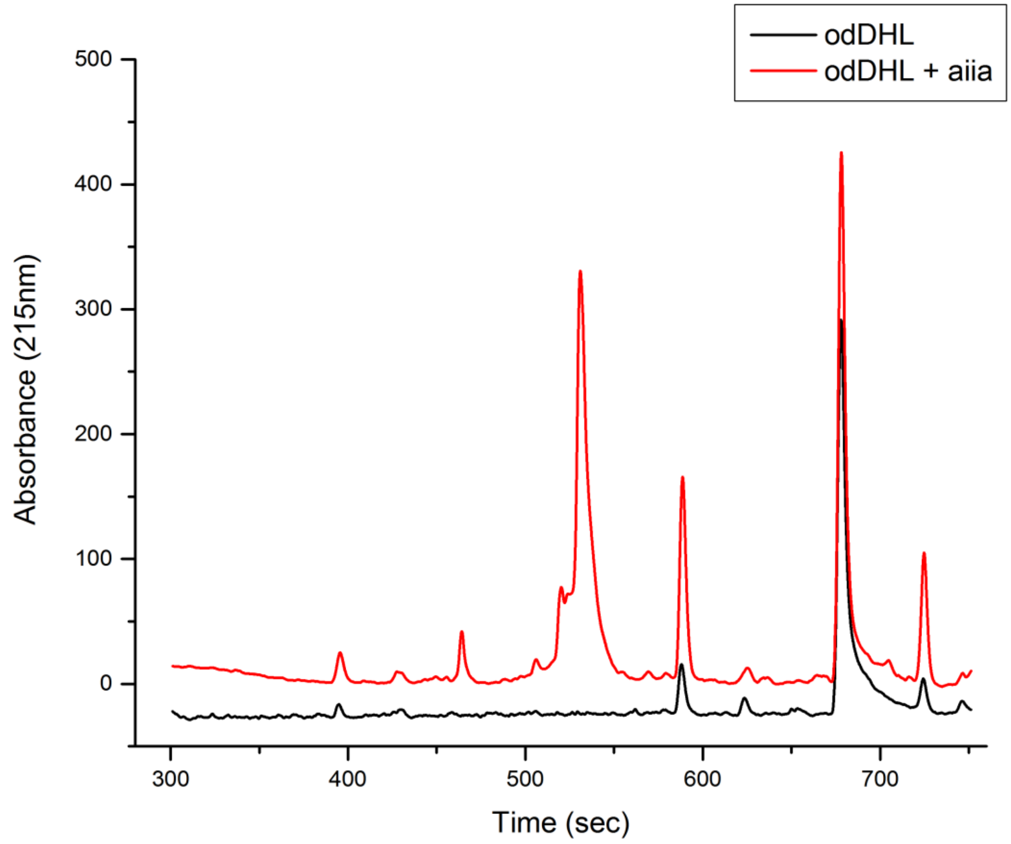

Supplementary Figure S8. HPLC analysis of enzymatic analysis of AiiA. The 3-oxo-C12-HSL (OdDHL) hydrolysis activity of AiiA was tested with 500 μM OdDHL and 30 μM AiiA in reaction buffer (20 mM Tris-HCl, 150 mM NaCl, pH 8.0). The hydrolyzed product of OdDHL could be observed as the read peak eluted earlier at t = 500 s.

REFERENCES

1. Kahlon RS. 2016. Pseudomonas: Molecular and Applied Biology.
2. Hmelo LR, Van Mooy BA. 2009. Kinetic constraints on acylated homoserine lactone-based quorum sensing in marine environments.
3. Fekete A, Kuttler C, Rothballer M, Hense BA, Fischer D, Buddrus-Schiemann K, Lucio M, Müller J, Schmitt-Kopplin P, Hartmann A. 2010. Dynamic regulation of N-acyl-homoserine lactone production and degradation in Pseudomonas putida IsoF. FEMS microbiology ecology 72:22-34.
4. Weber M, Buceta J. 2013. Dynamics of the quorum sensing switch: stochastic and non-stationary effects. BMC systems biology 7:6.
5. Goryachev AB, Toh DJ, Lee T. 2006. Systems analysis of a quorum sensing network: Design constraints imposed by the functional requirements, network topology and kinetic constants. Biosystems 83:178-187.
6. Régnier P, Arraiano CM. 2000. Degradation of mRNA in bacteria: emergence of ubiquitous features. Bioessays 22:235-244.
7. Fagerlind MG, Rice SA, Nilsson P, Harlén M, James S, Charlton T, Kjelleberg S. 2004. The role of regulators in the expression of quorum-sensing signals in Pseudomonas aeruginosa. Journal of molecular microbiology and biotechnology 6:88-100.
8. Nath K, Koch AL. 1971. Protein degradation in Escherichia coli II. Strain differences in the degradation of protein and nucleic acid resulting from starvation. Journal of Biological Chemistry 246:6956-6967.
9. Kaufmann GF, Sartorio R, Lee S-H, Rogers CJ, Meijler MM, Moss JA, Clapham B, Brogan AP, Dickerson TJ, Janda KD. 2005. Revisiting quorum sensing: discovery of additional chemical and biological functions for 3-oxo-N-acylhomoserine lactones. Proceedings of the National Academy of Sciences of the United States of America 102:309-314.
